# Characteristics of the *a* sequence of the duck Plague virus genome and specific cleavage of the viral genome based on the *a* sequence

**DOI:** 10.1186/s13567-023-01256-9

**Published:** 2024-01-03

**Authors:** Qiao Yang, Yaya Feng, Yuanxin Zhang, Mingshu Wang, Renyong Jia, Dekang Zhu, Shun Chen, Mafeng Liu, Xinxin Zhao, Ying Wu, Shaqiu Zhang, Bin Tian, Xumin Ou, Sai Mao, Juan Huang, Qun Gao, Di Sun, Zhen Wu, Yu He, Ling Zhang, Yanling Yu, Anchun Cheng

**Affiliations:** 1 Engineering Research Center of Southwest Animal Disease Prevention and Control Technology, Ministry of Education, Chengdu, 611130 China; 2 Key Laboratory of Animal Disease and Human Health of Sichuan Province, Sichuan Agricultural University, Wenjiang, Chengdu City, 611130 Sichuan China; 3 Avian Disease Research Center, College of Veterinary Medicine, Sichuan Agricultural University, Wenjiang, Chengdu City, 611130 Sichuan China

**Keywords:** Duck plague virus, *a* sequence, cleavage and packaging of viral genome, TaqMan dual qRT‒PCR

## Abstract

During the replication process, the herpesvirus genome forms the head-to-tail linked concatemeric genome, which is then cleaved and packaged into the capsid. The cleavage and packing process is carried out by the terminase complex, which specifically recognizes and cleaves the concatemeric genome. This process is governed by a cis-acting sequence in the genome, named the *a* sequence. The *a* sequence and genome cleavage have been described in some herpesviruses, but it remains unclear in duck plague virus. In this study, we analysed the location, composition, and conservation of *a* sequence in the duck plague virus genome. The structure of the DPV genome has an *a* sequence of (DR4)m-(DR2)n-*pac1*-S termini (32 bp)-L termini (32 bp)-*pac2*, and the length is 841 bp. Direct repeat (DR) sequences are conserved in different DPV strains, but the number of DR copies is inconsistent. Additionally, the typical DR1 sequence was not found in the DPV *a* sequence. The *Pac1* and *pac2* motifs are relatively conserved between DPV and other herpesviruses. Cleavage of the DPV concatemeric genome was detected, and the results showed that the DPV genome can form a concatemer and is cleaved into a monomer at a specific site. We also established a sensitive method, TaqMan dual qRT‒PCR, to analyse genome cleavage. The ratio of concatemer to total viral genome was decreased during the replication process. These results will be critical for understanding the process of DPV genome cleavage, and the application of TaqMan dual qRT‒PCR will greatly facilitate more in-depth research.

## Introduction

 Usually, the genome of a herpesvirus consists of a unique long (UL) region and a unique short (US) region, which are flanked by an internal repeat sequence (IRS) and a terminal repeat sequence (TRS), respectively [1, 2]. Upon entry into the nucleus, the genome is linked head-to-tail to form a cyclized genome, which is used as a template for replication to produce concatemeric genomes linked in a head-to-tail manner. The specific signal for genome cleavage is located in the *a* sequence of the genome termini called *pac1* and *pac2*, which contain all the cis-acting sequences needed for genome maturation [3–5]. In HSV-1, the *pac1* motif often contains 3–7 bp A- or T-rich regions flanked by poly (C) bundles, and the *pac2* motif consists of 5–10 bp A-rich regions and the GC region located at the distal end of *pac2*. After recognizing the cis-acting sequence of the concatemer by the terminase complex, the concatemer is pulled to the capsid portal composed of the pUL6 complex, which is the channel for genome import and exit [6–9]. After unit-length genome packaging is completed, pUL17, pUL25, and pUL36 form a capsid vertex-specific complex (CVSC) at the capsid vertex to seal the portal channel and prevent DNA leakage [10–14].

The genome type of HSV-1, a typical herpesvirus, is type E, and the arrangement is TRL-UL-IRL-IRS-US-TRS [15]. The herpes simplex virus 1 (HSV-1) sequence is repeated directly at both ends of the genome, UL and US, and exists in opposite directions at the UL and US junctions, with multiple copies at the UL end and the UL and US junction but only one copy at the US end [16–18]. The *a* sequence of HSV-1 contains three direct repeat sequences, designated DR1, DR2 and DR4. The structural pattern of the HSV-1 *a* sequence is DR1-*pac1*-(DR4)m-(DR2)n-*pac2*-DR1 with a length of 280 bp. DR1, DR2, and DR4 are 20, 12, and 37 bp direct repeat sequences, respectively. There are 19–22 copies of DR2 and 2–3 copies of DR4. DR1 is located on both sides of each sequence, and adjacent sequences share the same DR1 [5]. Usually, viral genome cleavage occurs in DR1, but the homology of DR1 in the *a* sequence of different herpesviruses is low [19].

Duck plague virus (DPV), also known as duck enteritis virus (DEV) or Anatid herpesvirus 1 (AnHV-1), is a member of *Mardivirus* in the *Alphaherpesvirinae* subfamily [20–22]. The DPV genome is a linear double-stranded DNA of approximately 158 to 162 kb. The DPV genome belongs to type D, which has only inverted repeat sequences (IRS, TRS) on either side of the US, forming the UL-IRS-US-TRS arrangement pattern, similar to that of the VZV genome [23]. Although the genome arrangement pattern of DPV and its genome organization have been reported, the exact structure of the genome termini containing cleavage packaging sites has remained largely unknown [24, 25]. In this study, we found that there are repeat sequences of 40 and 68 bp in length at the end of the duck plague virus genome. The copy number of repeat sequences varies during genome replication and between different duck plague virus strains. According to the genome sequence of the DPV CHv strain, the *a* sequence is located at 161 416 ~ 81 bp between US and UL in the duck plague genome, with a length of 841 bp, and the cleavage site is located between the positions of *pac1* and *pac2* in the *a* sequence. We also used Southern blotting and qRT‒PCR to detect cleavage of the DPV genome. It was found that the DPV genome is specifically cleaved at the genomic terminal sequence.

## Materials and methods

### Cell culture

Duck embryo fibroblasts (DEFs) were maintained in minimal essential medium (MEM, Gibco, Meridian Road Rockford, USA) supplemented with 10% foetal bovine serum (FBS), 100 U penicillin per mL, and 100 µg streptomycin per mL and cultured in a 37 °C incubator as described previously [26].

### Plasmid construction

To determine the DPV genomic concatemer terminal junction (CTJ) sequence, plasmids containing the CTJ sequence were generated. DEFs were infected with the wild-type DPV CHv strain at an MOI of 1 PFU per cell, and viral DNA was extracted at 48 h post-infection through phenol‒chloroform extraction. The CTJ fragment, 160 863–1445 bp, was obtained by PCR from viral DNA using the primers shown in Table 1 and was ligated into the BamHI- and EcoRI-restricted pUC118 vector (Takara, Beijing, China). Finally, 25 positive clones were obtained and sequenced.

**Table 1:** **Primers used in this study**

Primers	Primer sequence (5′–3′)	Function
pUC118-CTJ1-F/R	F:TATGACCATGATTACGAATTCGCCCAATTTCCCCAGTGTTATCCTGTG R:CAGGTCGACTCTAGAGGATCCTCTGAGGGAGGGAAGAAGAGT	To amplify the CTJ sequence for analysis of the progeny genome DR motif
pUC118-CTJs-F/R	F:ATGACCATGATTACGAATTCTGGTGTATTGCCAATCCTTTGCCGA R:AGGTCGACTCTAGAGGATCCGGGCGTGAAATACAAACTTGAGTTT	To construct the standard pUC118-CTJ for TaqMan dual quantification PCR
pUC118-UL31 s-F/R	F:TATGACCATGATTACGAATTCATTCAATTTCGCTCGTCTCA R:CAGGTCGACTCTAGAGGATCCCTTGTACCCAGGGATGCCAC	To construct the standard pUC118-UL31 for TaqMan dual quantification PCR
pTerS-F/R	F:CAATCCAGATGCATAGTCCGCGTG R:GAGACGGGACTCCGAAGGTAGT	To amplify S-terminus probe for southern blot
Qpcr-CTJ-F/R	F:GGTGGAGTTGGCATGTTG R:CTTCCATAGCAGTGCATTGA	To amplify CTJ sequence for TaqMan dual quantification PCR
Qpcr-UL31-F/R	F:CCATGAGAGCCAGATCTTC R:CTCCCGTACTATGGCTAAC	To amplify UL31 gene for TaqMan dual quantification PCR

### Sequence analysis of the DPV genomic terminal junction

To analyse whether there are differences between the genome sequences of progeny DPV, 25 CTJ sequences and the DPV genomic terminal sequence published in NCBI (GenBank No. JQ647509.1) were aligned using MUSCLE in MEGA [27]. Tandem repeat region analysis of CHv genome termini was performed by Tandem Repeats Finder [28]. The sequences of different DPV strains, CHv strains (GenBank No. JQ647509.1), VAC strains (GenBank No. EU082088), 2085 strains (GenBank No. JF999965), C-KCE strains (GenBank No. KF263690), 2013 strains (GenBank No. KF487736) and CV strains (GenBank No. KU216226), were also compared. Additionally, *a* sequence alignment between DPV and other herpesviruses, HSV-1 (GenBank No. NC_001806), GPCMV (GenBank No. NC_020231), HHV-3 (GenBank No. NC_001348), HHV-4 (GenBank No. AP015015), HHV-5 (NC_006273) and HHV-7 (NC_001716), was performed to analyse similarity.

### Southern blot analysis

DEFs were infected with DPV at an MOI of 10, and the cells were harvested at 12 h, 24 h, 36 h, 48 h, and 60 h post-infection. Total DNA was isolated and digested with BamHI for 8 to 10 h at 37 °C. The digested DNA was separated by 0.8% agarose gel electrophoresis, and then the agarose gel was soaked in 0.4 HCl for 15 min, neutralized with transfer buffer (NaCl 58.44 g/L, NaOH 10 g/L) for 15 min, and blotted onto a charged nylon membrane for 10–16 h. To detect cleavage of the DPV genome, a 431 bp S-terminus probe was generated by PCR using the primers pTerS-F and pTerS-R (Table 1) and labelled with biotin using a Thermo North2South™ Biotin Random Prime Labelling Kit (Thermo Fisher, Code No. 17 075). The probe pTerS recognizes the sequences at the IRS 124 151~124 581 bp and the TRS 161 984~161 414 bp. The membrane was exposed to UV light for 5 min and hybridized with a labelled probe at 55 °C for 12 h. The result was detected according to the instructions of Chemiluminescent Nucleic Acid Detection Module Kit (Thermo Fisher, Code No. 89 880).

### TaqMan dual real-time PCR

Copies of the genome concatemer and total genome were obtained using the qPCR-UL31 and qPCR-CTJ TaqMan probes (Table 2). The copies of the UL31 gene represent the total number of viral genomes, and the copies of CTJ represent the concatemeric genome. The TaqMan dual real-time PCR protocol included 0.6 µL qPCR-UL31-F/R primers (20 pmol/L), 0.5 µL qUL31 probe (10 pmol/L), 0.5 µL qPCR-CTJ-F/R primer pair (20 pmol/L), 0.6 µL qCTJ probe (10 pmol/L), Premix Ex Taq™ (TaKaRa, Code No. RR390Q) 10 µL, viral DNA 2 µL and H_2_O 5.8 µL. The reaction program was as follows: 95 °C for 20 s and 40 cycles of 95 °C for 5 s nd 60.6 °C for 20 s. The experiment was performed 3 times.

**Table 2:** **Probes used in this study**

Probes	Probes sequence (5′–3′)	5′-tag	3′-tag
QCTJ-P	CAAGCCACGCCCCTTTTGGC	VIC	MGB
QUL31-P	CGTACCGTACTGGCGACCGT	FAM	MGB

## Results

### Analysis of the DR sequence of DPV genome termini

Herpesviruses usually consist of a DR sequence and a *pac* sequence. Therefore, we first analysed the characteristics of the DR sequence of the DPV genome termini. According to the DPV genome published in NCBI, acting as a parent strain, primers were designed to amplify the concatemeric terminal junction sequence and perform DR sequence analysis.

**Figure 1:**
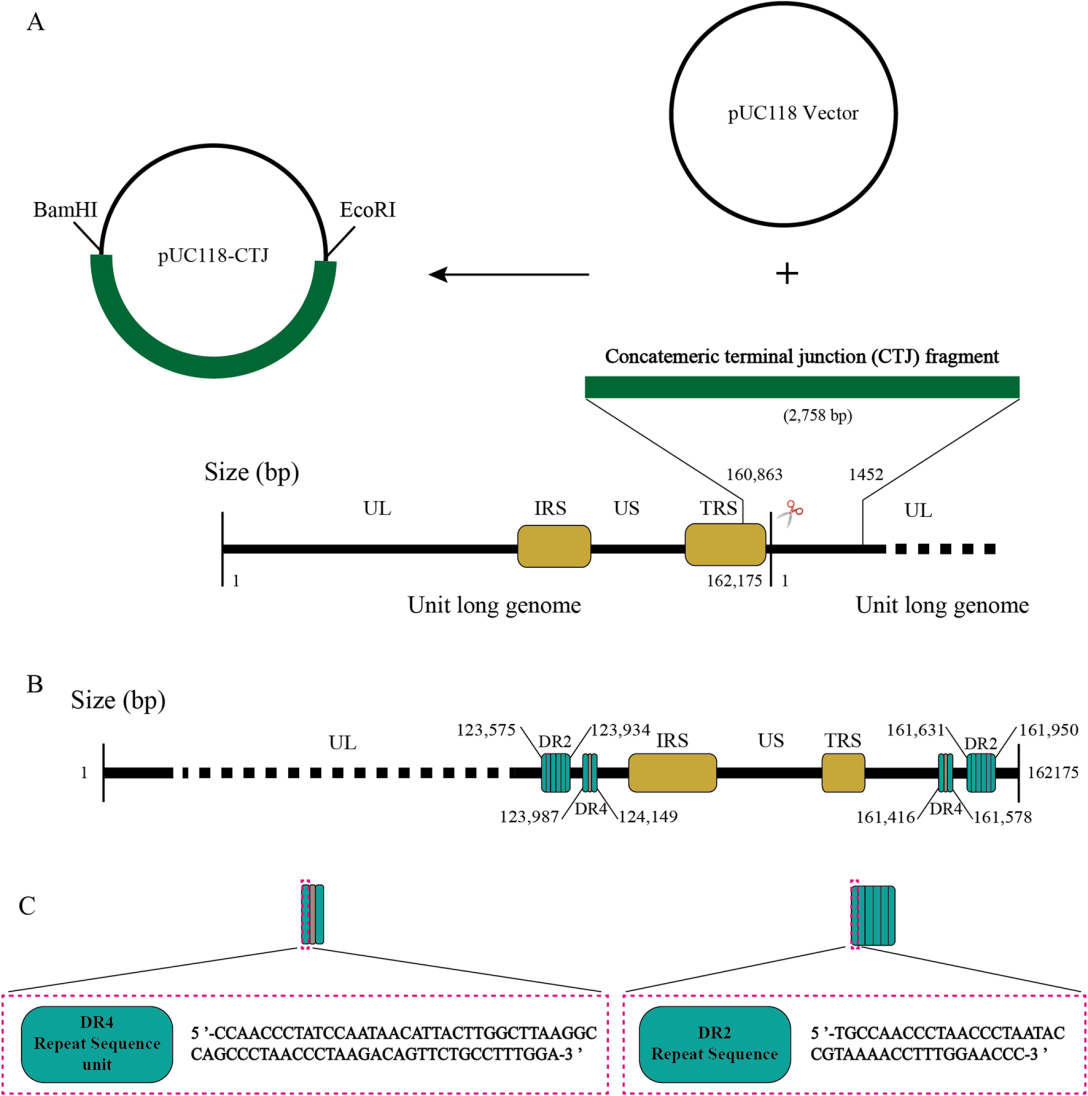
**The position of the DR sequence in the DPV genome. A** pUC118-CTJ is a plasmid carrying the DPV genome concatemeric terminal junction fragment, which is 2758 bp in length. The length of the monomeric DPV genome is 162 715 bp, and the cleavage site is located between the two adjacent genomes. **B** The position of DR sequences in the DPV genome. Each green square represents a DR repeat sequence unit, and the brown square between the two green squares represents a nonrepeating sequence between DR4 repeat units. **C** The sequence of DR4 and DR2 repeat units.

Twenty-five positive clones, named CTJ 1 ~ CTJ 25, were obtained and aligned to the parental CTJ sequences (160 863 bp~1445 bp) (Figure 1A). Sequence alignment indicated that most of the 26 CTJ fragments are conserved, with a similarity of 92.94%. The distribution of DR sequences in the 26 CTJ sequences was analysed using The Tandem Repeats Finder program. Two types of tandem repeat sequences, short and long repeat sequences, were identified, and the repeat units contain 40 and 68 bp, respectively (Figures 1B and C). However, the length of the CTJ sequences was found to be divergent between different CTJ clones because the number of copies of the DR sequence varies greatly (Table 3). The copy number of the repeat short sequences, identified as DR2, fluctuates widely from 0 to 9. Fourteen of the 26 CTJ sequences have 7 copies of DR2, accounting for the highest proportion of 53.85%. The details of different copy types are shown in Table 3, and it is worth noting that one of the CTJ clones does not contain DR2. Unlike DR2, DR4, the long repeat sequence, has only three types of copy numbers, 2, 1, and 0 copies. Two copies of DR4 occupy 92.30%. Interestingly, the CTJ sequence, which lacks DR2, also does not contain DR4. According to the results, the number of DR sequences in the progeny genome varies during replication, and the change in the number of copies does not affect viral genome replication.

**Table 3:** **The number of DR motifs at DPV genome termini**

DR motif	Copy number	Number	Proportion (%)
DR2 (40 bp)	NA	1/26	3.85
	5	1/26	3.85
	6	1/26	3.85
	7	14/26	53.85
	8	7/26	26.92
	9	2/26	7.69
DR4 (68 bp)	NA	1/26	3.85
	1	1/26	3.85
	2	24/26	92.30

To analyse the variation in DR sequences among different duck plague virus strains, CHv, VAC, 2085, C-KCE, 2013 and CV strains were chosen, and the genome terminal sequences of the six strains were compared (Table 4). The results showed that repeat units of terminal sequences in 6 strains are highly conserved. There are also two types of direct repeat units, 40 and 68 bp repeat units, in the genome termini of 6 different duck plague virus strains. There are three types of copy numbers in the DR2 sequences, 1, 6 and 8 copies. The CHv, VAC, 2013 and CV strains contain eight copies of the DR2 sequence, and the 2085 and CKCE strains have 1 and 6 copies of the DR2 sequence, respectively. The CTJ sequences in the 6 DPV strains all contain two copies of DR4. According to the above results, the direct repeat sequences among different DPV strains or progeny DPV genomes are highly uniform. Interestingly, DPV tends to produce genomes with more DR sequences in a particular range. We could not find the DR1 sequence in the DPV genome termini.

**Table 4:** **The number of DR motifs in different duck plague strains**

DR motif	Copy number	Number of DPV strain/Total number of DPV strain	Proportion (%)
40 bp TR	1	1/6	16.67
	6	1/6	16.67
	8	4/6	66.67
68 bp TR	2	6/6	100.00

### Characteristics of the ***pac1*** and ***pac2*** sequences

**Figure 2:**
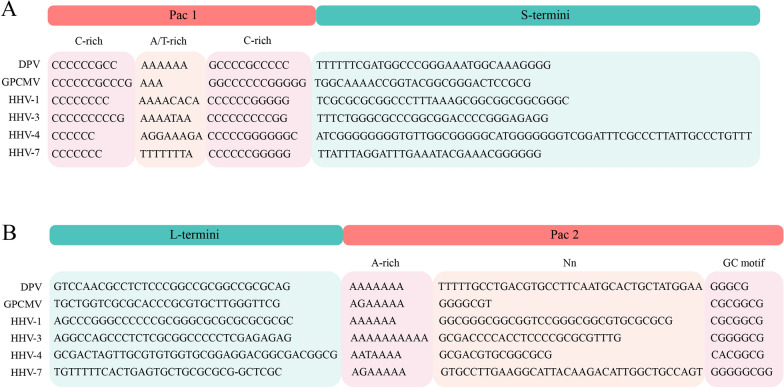
**Sequence analysis of the DPV pac element with its homologues. A** The *pac1* motif at the S-terminus contained three characteristic regions, including an A/T-rich region in the middle of *pac1* and two C-rich regions on both sides of the A/T-rich region. **B** The *pac2* motif is located at the L-terminus and consists of three regions: the A-rich region, disordered Nn region and CG motif region.

*Pac1* and *pac2* sequences, which guide cleavage and packaging of the herpesvirus genome, have been shown in other herpesviruses [29–31]. The sequences and locations of *pac1* and *pac2* are conserved in herpesviruses. According to the sequence alignment of genome termini between DPV and other herpesviruses, putative *pac1* and *pac2* sequences were identified in DPV CHv strain genome termini. The *pac1* motif is located at the S-terminal 162 118–162 143 bp and contains 26 base pairs, and the sequence of *pac1* is CCCCCCGCCAAAAAAGCCCCGCCCCC (Figure 2A). The *pac2* motif is located at the L-terminal 33–81 bp and includes 49 base pairs. The sequence of *pac2* is AAAAAAATTTTTGCCTGACGTGCCTTCAATGCACTGCTATGGAAGGGCG (Figure 2B). The *pac1* motif is composed of three characteristic regions, including an A/T-rich region located in the middle of *pac1* and two C-rich regions located on both sides of the A/T-rich region (Figure 2A). The *pac2* motif also consists of three regions: the A-rich region, disordered Nn region and CG motif region (Figure 2B). Except for the Nn region, the rest of *pac1* and *pac2* are conserved in different herpesvirus and DPV strains.

### The complete sequence of the duck Plague virus genome

**Figure 3:**
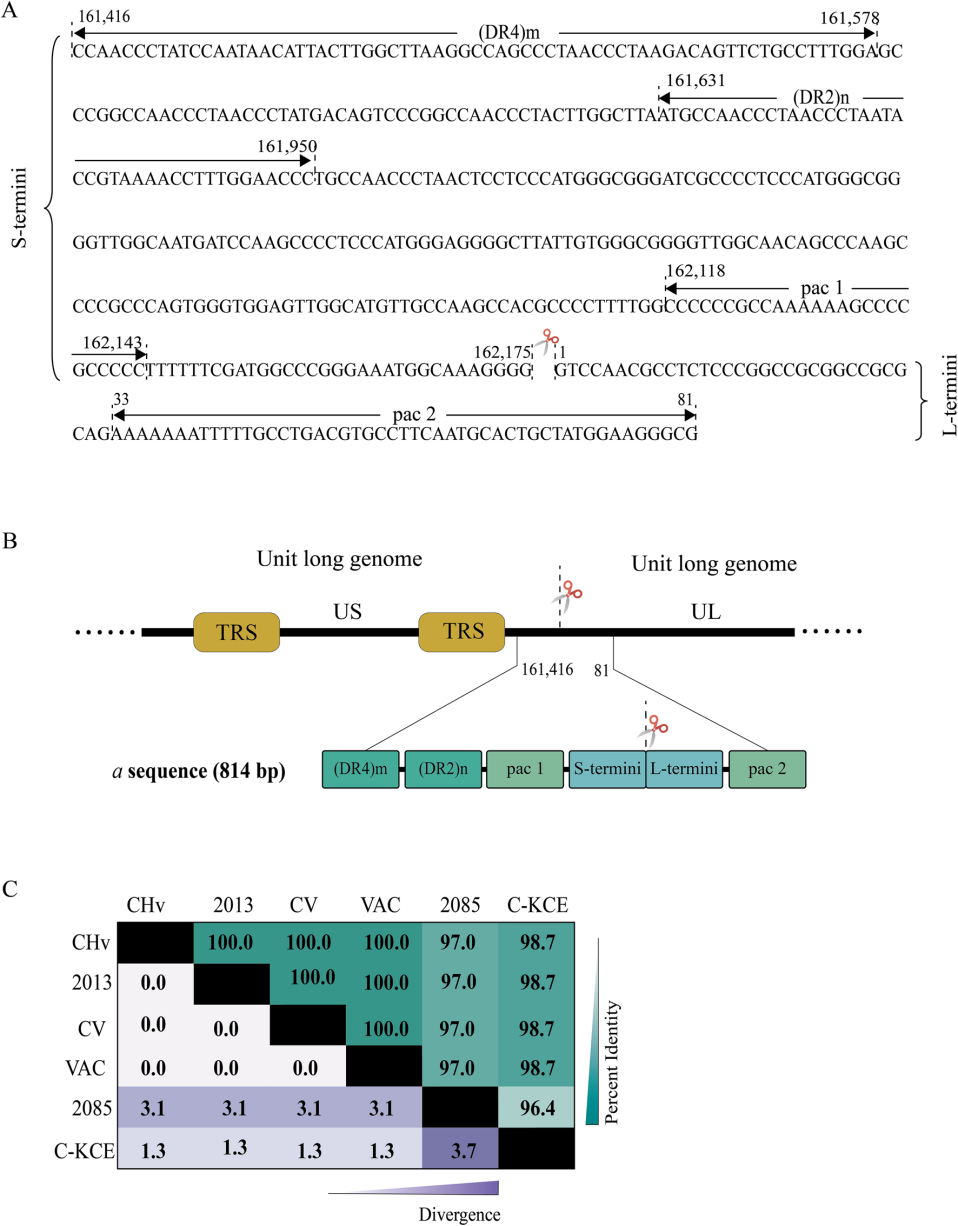
**Structure and conservation analysis of the a sequence of the DPV genome. A** The *a* sequence composition of the DPV genome. The scissor indicates the junction site of the S-termini and L-termini. Repeated long sequences (DR4), repeated short sequences (DR2) and *pac* sequences are marked. “m” represents the number of DR4, and “n” represents the number of DR2. **B** The position of the *a* sequence in the DPV genome. The *a* sequence is located at 161 416 ~ 81 bp between the US and UL of the adjacent DPV genomes, with a length of 841 bp. **C** Identity and divergence analysis of sequences in different DPV strains.

According to the DR motifs and the *pac* motif, we obtained the complete structure of *a* sequence of DPV, (DR4)m-(DR2)n-*pac1*-S termini (32 bp)-L termini (32 bp)-*pac2* (Figure 3A). The full-length sequence of the DPV CHV strain is 841 bp and located at 161 416 ~ 81 bp. Based on alignment of *a* sequence between DPV and other herpesvirus genome sequences, a significant difference between DPV and HSV-1 was found. There are complete sequences in the HSV-1 monomeric genome, and each end of the genome has at least one sequence. However, there is no complete sequence structure in the DPV monomeric genome, and it only has the complete sequence at the junction of the concatemeric genome (Figure 3B). In the DPV genome, there are six motifs in *a* sequence: DR4 motif, DR2 motif, *pac1* motif, 32 nucleotides of S termini, 32 nucleotides of L termini and *pac2* motif (Figure 3A). (DR4)m-(DR2)n*-pac1*-S termini (32 bp) located in the termini of TRS and L termini (32 bp)-*pac2* at the UL end can be spliced to form a complete sequence structure in the concatemeric genome. In the CHv genome, (DR4)m-(DR2)n-*pac1*-S termini (32 bp) is located at 161 416–162 175 bp, and there are inverted repeats in the genome from 123 350 to 124 149 bp that have one more copy than the terminal DR2 repeat. L termini (32 bp)- *pac2* is located at 1–81 bp at the L end of the viral genome, and L termini (32 bp) have another reverse copy sequence at 123 318–123 349 bp of the genome and are connected with S termini (32 bp) in the genome. However, there is only one copy of the *pac2* motif in the DPV genome, located at 33–81 bp at the L-terminus of the genome (Figure 3B). To further analyse conservation of the DPV *a* sequence in different strains, sequences of 6 DPV strains (CHv, 2013, CV, VAC, 2085, CKCE) were aligned using MEGA7.0.14. The *a* sequences among the strains are highly conserved, with similarity over 96.4% (Figure 3C).

### Specific cleavage of the DPV concatemeric genome

**Figure 4:**
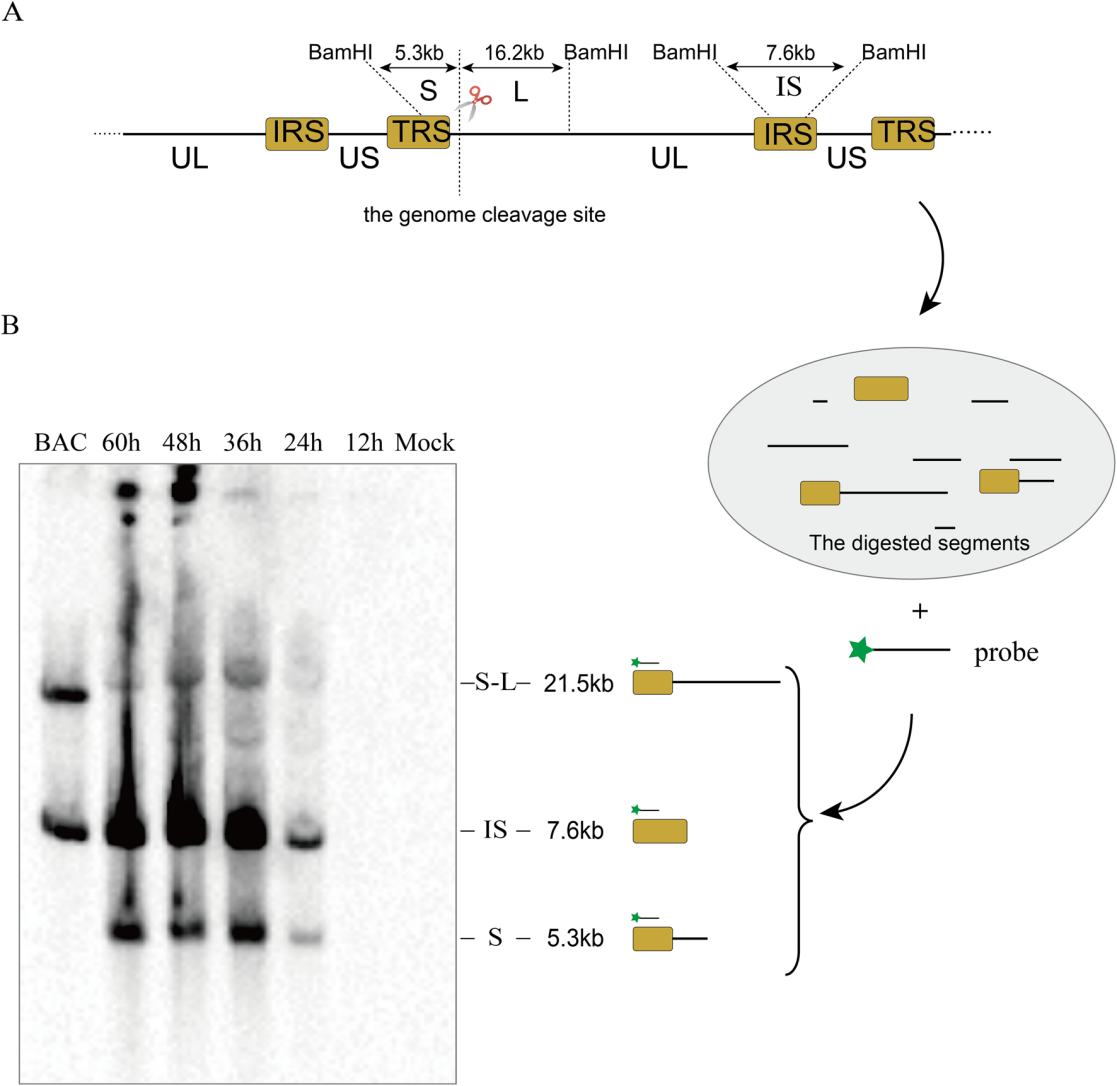
**Southern blot detection of DPV genome cleavage. A** Schematic diagram of Southern blotting. **B** The result of Southern blotting.

To determine whether DPV genomes form concatemers and are cleaved into monomeric genomes, the pTerS probe was designed to recognize concatemeric and monomeric genomes. The pTerS probe can identify the US termini of the concatemer and monomer and the inverted US termini inside the genome. After BamHI digestion of the total DPV genomes, three bands in each infected sample from 24 hpi to 60 hpi were detected. The US termini of monomers, named S, are approximately 5.3 kb. The band over 21.5 kb, S-L, is the spliced sequence of the S and L termini of adjacent genomes. The third band, IS, is approximately 7.6 kb and located in the inverted internal S-terminal sequence (Figures 4A, B). This result is consistent with our speculation that the DPV genome can form concatemers and is cleaved into monomers. The S segment represents the monomeric genome, and its amount increased with infection time. This result suggests that the mature genome of DPV increases in the later stages of infection. Similarly, the IS sequence represents the total DPV genome, containing the monomeric and concatemeric genomes, and continuously increased after 24 hpi. However, the amount of S-L segment displayed a slight rise during infection, which was obviously different from S and IS. It is speculated that formation and cleavage of concatemeric genomes are dynamic during viral replication, leading to a lack of accumulation of the concatemeric genome. Due to its circular morphology, the DPV BAC plasmid as a control showed only two bands (S-L and IS). Moreover, the bands of the S and S-L segments were specific during the process of genome cleavage. This result suggests that cleavage of the DPV concatemeric genome occurs at a specific site. In addition, there were no bands larger or smaller near the S or S-L, and it is also believed that the DR sequence in the sequence of the DPV genome mainly exist in one dominant copy type, even though different copy types were observed.

### Cleavage of the genome concatemer analysed using TaqMan dual qRT PCR

**Figure 5:**
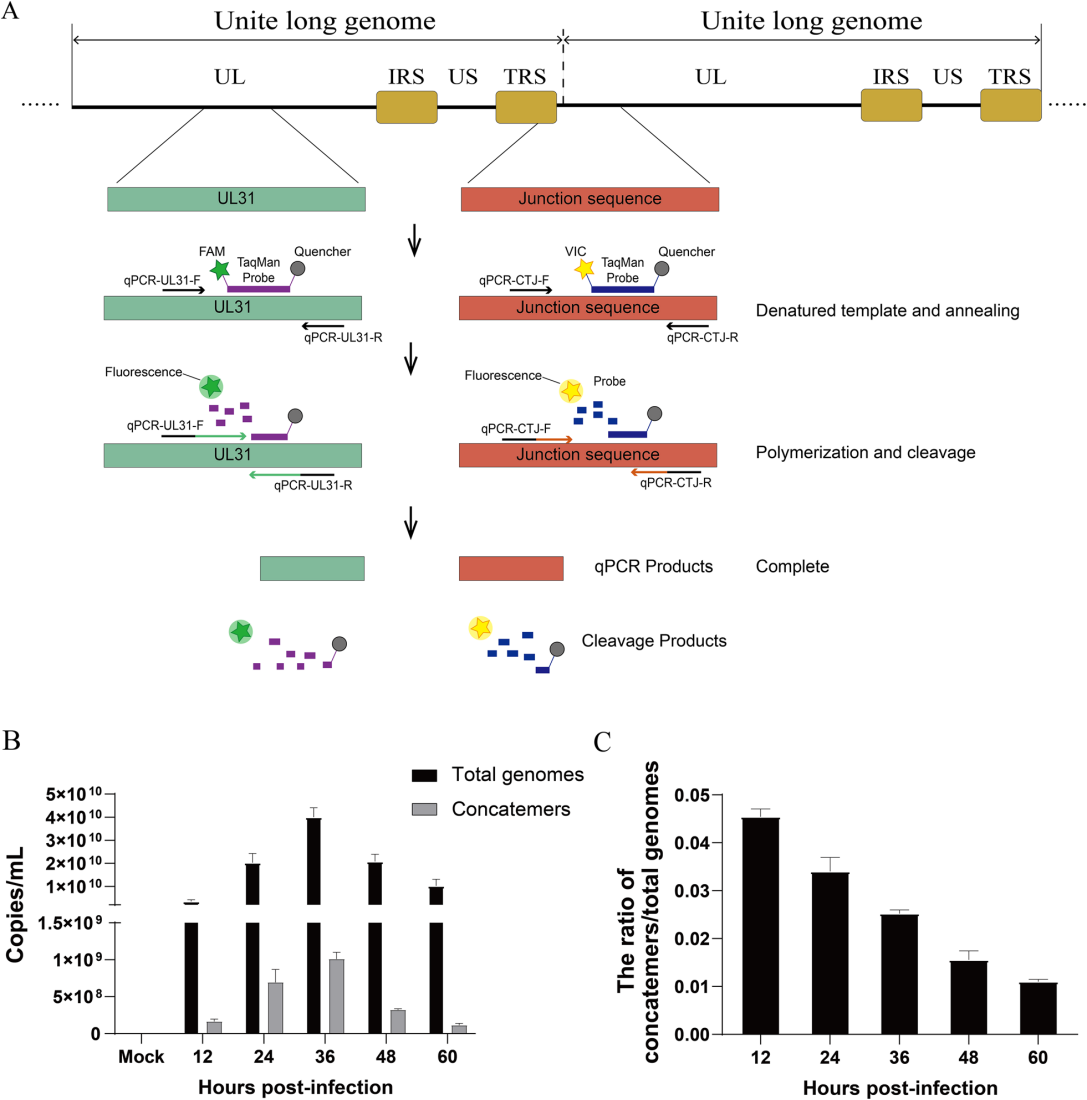
**TaqMan dual qRT‒PCR analysis of the dynamics of DPV genome cleavage. A** Schematic diagram of TaqMan dual qRT‒PCR. **B** Copies of total genomes and concatemers at different infection times. **C** The ratio of the copy number of concatemers to the total genome.

The TaqMan probe dual fluorescence quantitative real-time PCR was used to further investigate formation of the DPV genome concatemer and the dynamic process of cleavage. DNA was obtained in the same manner as the Southern blot assay. The total amount of DPV genome generated during replication was quantified using the probe of the UL31 gene. The amount of concatemeric genome was detected by the probe of the junction of adjacent genomes (Figure 5A). The change in cleavage was analysed by the ratio of the number of concatemers to the total number of genomes, and the results clarified the cleavage of concatemers.

The viral genome could be detected earlier because the qRT‒PCR method is more sensitive than Southern blotting. As shown in Figure 5B, the copy number of concatemeric genomes and the total copy number of the viral genomes both peaked at 36 hpi. After 36 hpi, the copy number of the concatemeric genome and the total copies of the viral genome decreased. It was possible that the infected cells detached from the cell culture plate during the later stage of infection. However, although the trends in the amount of concatemeric genome and total viral genome are consistent, the ratio between them decreased with the infection time. The highest ratio was at the beginning of infection, but the proportion of concatemers was still low, less than 5%, and gradually decreased from 4.54% at 12 hpi to 1.10% at 36 hpi (Figure 5C). It is suggested that many concatemers were cleaved into monomers from 12 hpi and that the cleavage rate gradually increased compared with the genome replication rate, indicating that the cleavage event of the genome improved during the replication process.

## Discussion

The *a* sequence is essential for herpesvirus genome maturation and mainly consists of DR1, DR2, DR4, *pac1* and *pac2* motifs [31–33]. However, there are usually different copy numbers in the genome with different repeat sequences in the same or different herpesvirus genomes. The DPV genome forms concatemers, and the L-terminus and S-terminus are covalently linked to form a unique and complete sequence. The arrangement of the DPV *a* sequence is (DR4)m-(DR2)n-*pac1*-S termini (32 bp)-L termini (32 bp)-*pac2.* In the DPV progeny viral genome, the copy numbers of DR2 and DR4 are variable, but the progeny genome with more copies of DR2 or DR4 is dominant. DPV has an *a* sequence different from that of other herpesviruses. The *a* sequence of the HSV-1 genome is arranged as DR1-*pac1*-(DR4)m-(DR2)n-*pac2*-DR1 [34, 35]. However, we could not find the DR1-like motif on either side of the *a* sequence or near *pac1* and *pac2* in the DPV genome. It is speculated that the DR1 sequence may not exist or only exists between the *pac*1 and *pac*2 motifs in the DPV *a* sequence. In HSV-1, there are multiple copies of sequences that are located in the TRL and TRS at the end of the genome as well as in the internal IRL and IRS [36–38], but there is only a complete copy of *a* sequence in the DPV junction adjacent genome. Although the DPV *a* sequence is different from that of HSV-1, it does not affect replication and cleavage of the DPV genome.

Southern blotting was first used to detect cleavage of the DPV genome. The results showed that unique TS, S-L and IS segments were produced after BamHI digestion but that they differed from those of HSV-1 and KSHV. After restriction enzyme digestion, segments of various sizes were produced at TRL and IRL-IRS of the HSV-1 genome due to the multiple copies of the *a* sequence [39, 40]. In KSHV, genome cleavage of KSHV occurs in the GC-rich terminal repeat (TR) sequence, generating TR sequences with fragments of different sizes and producing a ladder-like cleavage pattern; this indicates that the KSHV genome is variable with respect to the size of the TR fragment at the end of the monomeric genome produced during the cleavage and packaging process. This phenomenon differs significantly from DPV [41–43]. TaqMan dual fluorescence quantitative real-time PCR was also used to detect the dynamic cleavage process of DPV due to its higher sensitivity. Compared with the DPV total genome, it is evident that the copies of concatemers are always at a low level, which is consistent with the result of Southern blotting. This indicates that the concatemers are continuously cleaved into monomers during replication. Application of this method may be more simple and sensitive to detect cleavage of the viral genome, which will greatly facilitate detection of viral genome cleavage.

DPV is a member of the herpesvirus family, but the mechanism of viral genome cleavage and packaging is still unclear. In this study, we analysed the composition and sequence characteristics of the DPV genome, which can provide an essential theoretical basis for further analysis of genome cleavage and packaging processes. It not only proves the specificity of DPV genome cleavage using classical Southern blotting but also establishes a more sensitive TaqMan duel qRT PCR, which can make detection of the viral genome cleavage process more convenient. By using this method, we can further explore the function of each motif in the *a* sequence.

## Data Availability

The datasets used and/or analysed during the current study are available from the corresponding author upon reasonable request.
